# Methicillin-Resistant *Staphylococcus aureus* Nasal Colonization in Chinese Children: A Prevalence Meta-Analysis and Review of Influencing Factors

**DOI:** 10.1371/journal.pone.0159728

**Published:** 2016-07-21

**Authors:** Jialing Lin, Yang Peng, Ping Xu, Ting Zhang, Chan Bai, Dongxin Lin, Qianting Ou, Zhenjiang Yao

**Affiliations:** 1 Department of Epidemiology and Health Statistics, Guangdong Pharmaceutical University, Guangzhou, China; 2 Centre for Chronic Disease, University of Queensland, Brisbane, Australia; Kent State University, UNITED STATES

## Abstract

**Objective:**

To determine the pooled prevalence and review the influencing factors of methicillin-resistant *Staphylococcus aureus* (MRSA) nasal colonization in Chinese children.

**Methods:**

Articles published between January 2005 and October 2015 that studied prevalence or influencing factors of MRSA nasal colonization in Chinese children were retrieved from Chinese Biomedical Literature database (CBM), China National Knowledge Infrastructure (CNKI) database, Chinese VIP database, Chinese Wanfang database, Medline database and Ovid database. Prevalence and influencing factors were analyzed by STATA 13.1.

**Results:**

Thirteen articles were included. The overall prevalence of MRSA nasal colonization was 4.4% (95% confidence interval [CI]: 0.027–0.062). With an MRSA prevalence of 3.9% (95% CI: 0.018–0.061) in healthy children and 5.8% (95% CI: 0.025–0.092) in children with underlying medical conditions. Children recruited in the hospitals presented MRSA prevalence of 6.4% (95% CI: 0.037–0.091), which was higher than those recruited in the communities [2.7% (95% CI: 0.012–0.043)]. A number of influencing factors for MRSA nasal colonization were noted in three eligible studies: gender (male vs female; OR: 0.67; 95% CI: 0.55–0.82), younger age (OR: 2.98; 95% CI: 1.31–6.96 and OR: 1.56; 95% CI: 1.21–2.00), attending day care centers (OR: 2.97; 95% CI: 1.28–6.76), having infectious diseases (OR: 2.31; 95% CI: 1.10–4.52), using antibiotics (OR: 2.77; 95% CI: 1.45–5.05), residing in northern Taiwan (OR: 1.41; 95% CI: 1.15–1.71), passive smoking (OR: 1.30; 95% CI: 1.02–1.63), and pneumococcal vaccination (OR: 1.22; 95% CI: 1.01–1.48).

**Conclusions:**

Children could act as reservoirs of MRSA transmissions. Hospitals remained the most frequent microorganism-circulated settings. More MRSA infection control strategies are required to prevent the dissemination among children.

## Introduction

In the last decade, the overall burden of methicillin-resistant *Staphylococcus aureus* (MRSA) has considerably increased, both in communities and healthcare settings [1, 2]. It has caused serious health consequences since it was first identified in 1961 [3]. A number of studies have reported that MRSA can cause adverse clinical outcomes, including necrotizing pneumonia [4, 5], subcutaneous abscesses [6] and so on, which should raise our awareness of elucidating its current situation and taking relevant prevention strategies. In the United States, the proportion of methicillin resistance in *Staphylococcus aureus* (*S*. *aureus*) strains approached almost 60% in 2003, with an average resistance rate of around 50% over the period 1998–2002 [7]. In Europe, the proportion of methicillin resistance in *S*. *aureus* strains, which are isolated from infected patients, varied from less than 0.5% to more than 50% in 2011, with a pooled average rate of around 17% [8].

Children are important reservoirs of MRSA and may play a central role in disseminating MRSA in the community and hospital settings [9]. However, there is lack of data regarding the prevalence of MRSA among Chinese children due to inconsistent findings and limited sample sizes. In addition, the articles regard influencing factors of MRSA colonization are rare.

Hence, it is necessary to conduct a meta-analysis to comprehensively determine the prevalence and explore the influencing factors of MRSA nasal colonization in Chinese children, which may helpful to establish public health interventions to reduce MRSA infection.

## Materials and Methods

### Literature Database

This meta-analysis followed the Meta-analysis of Observational Studies in Epidemiology (MOOSE) guidelines and Preferred Reporting Items for Systematic Reviews and Meta-Analyses (PRISMA) guidelines (S1 Table). Major electronic databases were systematically searched. They were Chinese Biomedical Literature database (CBM), China National Knowledge Infrastructure (CNKI) database, Chinese VIP database, Chinese Wanfang database, Medline database and Ovid database. Key words used for search were: (“MRSA” OR “methicillin resistant *Staphylococcus aureus*”) and (“nasal” OR “nasopharyngeal”) and (“colonization” OR “carriage”) and “children”. In order to reflect the epidemiology of MRSA in recent years, we searched for articles on MRSA nasal colonization in children that were published between January 2005 and October 2015. References of all included articles for additional studies were scanned, either. No language restrictions were applied.

### Inclusion and Exclusion Criteria

To be included in this meta-analysis, studies must meet all the following criteria: (a) study design should be cross-sectional; (b) the subjects were proven Chinese children, which means those who resided in China; (c) provided total number and percentage of identified MRSA nasal colonization. Studies were excluded when they were: (a) based on a mixed adult-children population without specific data on the children population or reported epidemiological data on outbreaks; (b) microbiological studies of which subjectives were microbes but not humans; (c) duplicate reports; (d) reviews, letters, editorial articles or meta-analyses.

### Data Extraction

Data from the published studies were extracted independently by two reviewers. For each study, the following characteristics were collected: the first author, year of publication, study period, age, type of settings (community or hospital), presence of diseases and province. In case of conflicting evaluations, the disagreements were resolved by discussion among the whole group members.

### Quality Assessment

The quality of studies was assessed using a validated quality assessment tool for cross-sectional studies [10]. The following eight items were assessed to calculate a total quality score: (1) clear definition of the target population; (2) representative of probability sampling; (3) sample characteristics matching the overall population; (4) adequate response rate; (5) standardised data collection methods; (6) reliability of survey measures/instruments; (7) validation of survey measures/instruments; and (8) appropriate statistical methods. Answers were scored 0 or 1 for 'No' and 'Yes'. The total quality score varied between 0 and 8 for each study. Total scores of 0–4 and 5–8 were regarded as low and high quality, respectively. Two authors separately evaluated the quality scores of each study and any disagreement was settled by discussions of the whole group.

### Statistical Analysis

We performed a meta-analysis using random effects model (DerSimonian Laird method [11]) to obtain a pooled prevalence and corresponding 95% confidence interval (CI). Statistical heterogeneity between and within groups was estimated using Chi-square based Q statistic with a *P*-value<0.1 or *I*^*2*^>50% as statistically significant heterogeneity [12].

Subgroup analyses were conducted by type of settings (community and hospital), presence of underlying condition (healthy, atopic dermatitis, *S*. *aureus* infection, respiratory infection, and unclear), age range (non-neonates and neonates), region (mainland China, Taiwan, and Hong Kong), and study period (2001–2004, 2005–2010, and 2011–2014). Influencing factors were analyzed by Odds Ratios (ORs) and 95% CIs.

The funnel plot, Begg’s rank correlation test [13], and Egger’s linear regression test [14] were introduced to assess the publication biases, with *P*<0.1 indicating potential bias. In addition, sensitivity analysis was applied to assess the influence of each individual study. For subgroup analyses, the significance of the overall effect was calculated by Z test. A Z score acted as the ratio of the overall effect to its standard error and can compare its standard error with the standard normal distribution. The pooled prevalence, Begg’s rank correlation test, Egger’s linear regression test, and sensitivity analysis were conducted by the STATA (Version 13.1).

## Results

### Characteristics of the Eligible Studies

**Fig 1:**
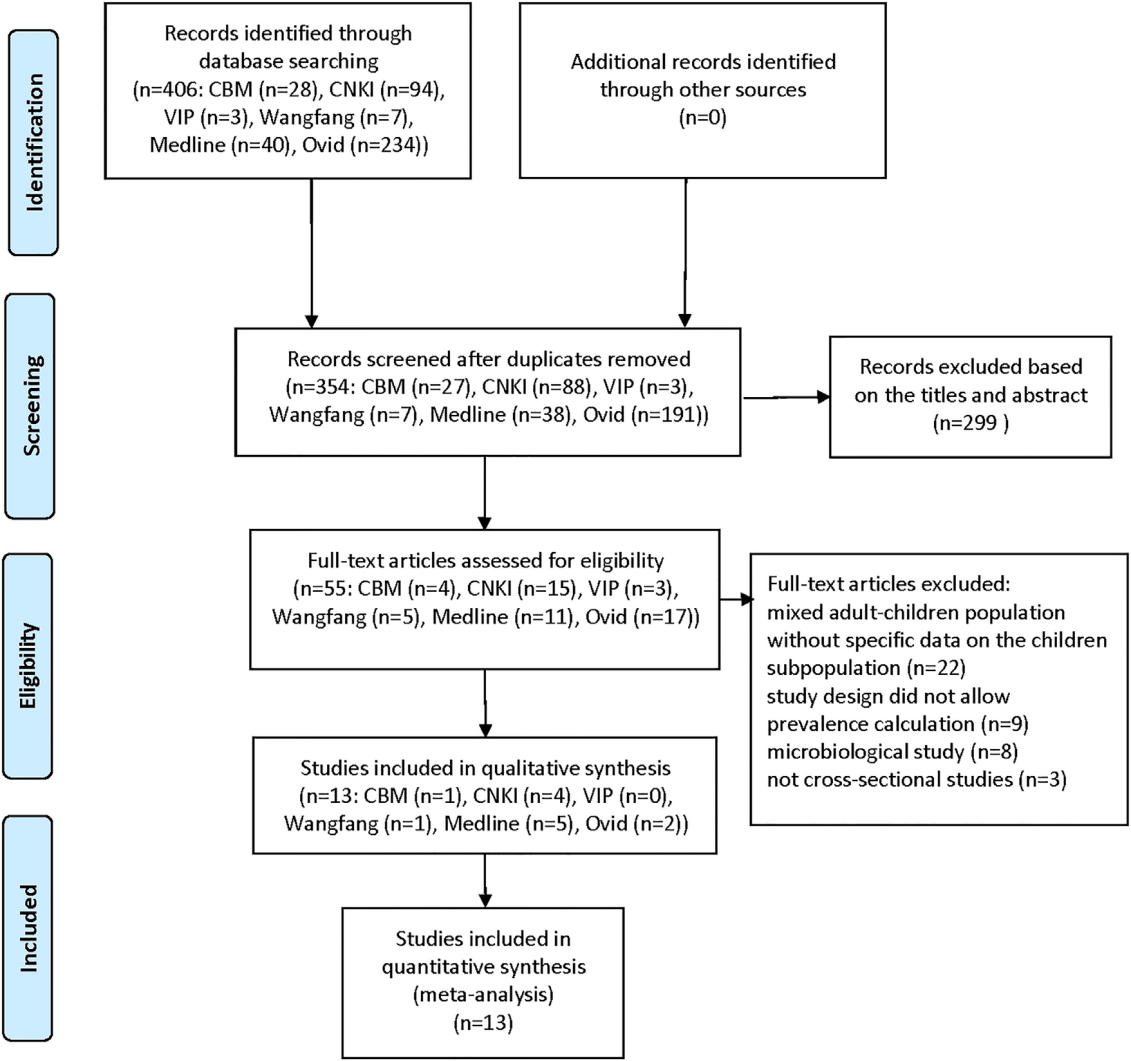
Flow diagram of the selection process of the included studies.

The process of study selection is shown in Fig 1. Thirteen studies were included [15–27]. Thirteen and three of them reported the nasal colonization prevalence and reviewed the influencing factors, separately. Identification of MRSA in the 13 eligible studies was different, five studies were based on the *mecA* gene [15, 17, 20, 22, 23], five were cefoxitin disk diffusion method [16, 18, 19, 21, 24], and three were oxacillin disk diffusion method [25–27]. Six articles were Chinese and seven articles were English. The main characteristics of the included studies (first author, publication year, study period, kind of setting, age, location, presence of underlying condition, population, and prevalence) are reported in Table 1. In addition, the results of quality assessment were also reported in Table 1. The mean quality score of the 13 eligible studies was 7 (range, 6–8). In the 13 studies, six of the studies were reported in communities (day-care centers or schools) [16, 18, 19, 23, 25, 26] and seven studies were reported data from hospitals [15, 17, 20–22, 24, 27], in which one study reported data from a neonatal ICU [17].

**Table 1:** Characteristics of selected studies.

First author, publication year	Study period	Location	Setting	Age means, range	Presence of underlying condition	Sample size	MRSA prevalence (95% Confidence Interval)	Quality scores of studies	References
Shan Tan, 2015	2013–2014	Sichuan	Hospital	-, 0-18y	*S*. *aureus* infection	56	0.125 (0.038–0.212)	6	[15]
Jinjian Fu, 2015	2011	Guangdong	Community	8.7, 2.5-12y	Healthy	1448	0.019 (0.012–0.026)	6	[16]
Hongxiang Guo, 2013	2011–2012	Henan	Hospital	5.9d, 0-01m	Unclear	1678	0.037 (0.028–0.046)	7	[17]
Pakleung Ho, 2012	2009–2010	Hong Kong	Community	3.9, 2-5y	Healthy	2211	0.013 (0.008–0.017)	8	[18]
Jianjun Deng, 2012	-	Sichuan	Community	-, 2-18y	Healthy	2373	0.068 (0.058–0.078)	7	[19]
Jianjun Deng, 2012	-	Sichuan	Hospital	-, 0-16y	Respiratory infection	315	0.010 (-0.001–0.002)	7	[20]
Chihjung Chen, 2011	2005–2008	Taiwan	Hospital	25, 2-60m	Healthy	6057	0.078 (0.071–0.085)	8	[21]
Chingshen Tang, 2011	2005–2009	Taiwan	Hospital	-, 0-18y	Atopic dermatitis	188	0.191 (0.135–0.248)	7	[22]
Juan Fan, 2011	2005	Sichuan	Community	4, 2-7y	Healthy	801	0.011 (0.004–0.019)	7	[23]
Yhuchering Huang, 2007	2005–2006	Taiwan	Hospital	-, 2-5y	Healthy	3046	0.073 (0.063–0.082)	8	[24]
Poliang Lu, 2005	2001	Taiwan	Community	-, 2-18y	Healthy	987	0.033 (0.022–0.045)	6	[25]
Yhuchering Huang, 2005	2001–2002	Taiwan	Community	-	Healthy	262	0.019 (0.003–0.036)	8	[26]
KLE Hon, 2005	2004	Hong Kong	Hospital	-	Atopic dermatitis	55	0.018 (-0.017–0.053)	6	[27]

### Population Characteristics

According to the presence of underlying condition, five articles were based on a population of sick children [15, 17, 20, 22, 27] and the remaining were based on healthy population [16, 18, 19, 21, 23–26]. Among five articles regarding sick population, two were atopic dermatitis [22, 27], one was *S*. *aureus* infection [15], one was respiratory infection [20], and one was unclear [17]. Most of the studied populations were >100. With regard to age range, 11 of the studies were included in the review, which was reported in Table 1.

### Pooled MRSA Prevalence

In the 13 articles included in the meta-analysis, we observed an MRSA nasal colonization prevalence ranging from 1.0% to 19.1%. And the number of children included in the 13 articles was 19477.

**Fig 2:**
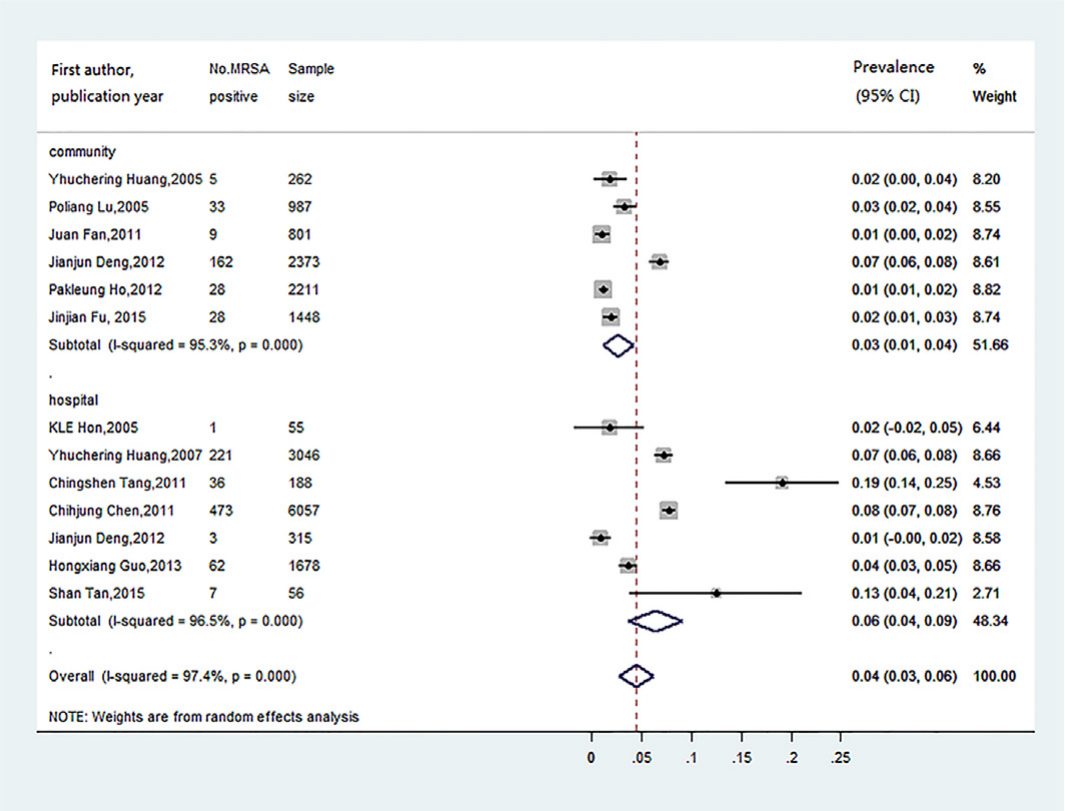
Forest plot for MRSA prevalence and 95% CI for all selected studies. (A) Pooled prevalence estimate of MRSA using random effect. (B) ES: effect size. (C) CI: confidence interval.

There was a significant heterogeneity among the 13 studies (χ^2^ = 467.17; *P*< 0.001; *I*^*2*^ = 97.4%). Thus, the random effect method was used to obtain pooled MRSA nasal colonization prevalence (4.4%, 95% CI: 2.7%-6.2%), which was reported in Fig 2. Subgroup analyses were conducted by settings, presence of underlying conditions, age range, region, and study period. All the pooled MRSA prevalence and corresponding 95% CIs of subgroups were obtained, which were reported in Table 2. Among these subgroups, heterogeneity did still exist, except in the group *S*. *aureus* infection in presence of underlying conditions, group Hong Kong in region, and group 2011–2014 in study period. The pooled prevalence of MRSA nasal colonization was significantly higher (*P* for difference< 0.001) in hospitals than that in communities. Significant differences were also found across age ranges (*P* for difference = 0.001), presence of underlying conditions (*P* for difference< 0.001), and regions (*P* for difference< 0.001). No significant differences were noticed among subgroups classified by study period.

**Table 2:** Prevalence estimates by subgroups.

Subgroups	Number of studies	MRSA prevalence	95% Confidence Interval		*P*^a^	*P*^b^	*I*^*2*^ (%)
**Settings**							
Community	6	0.027	0.012	0.043	0.001	<0.001	95.3
Hospital	7	0.064	0.037	0.091	<0.001		96.5
**Presence of underlying condition**							
Healthy	8	0.039	0.018	0.061	<0.001	<0.001	98.3
Atopic dermatitis	2	0.103	-0.066	0.273	0.005		96.2
*S*. *aureus* infection	1	0.125	0.138	0.212	0.233		-
Respiratory infection	1	0.010	-0.001	0.020	0.082		-
Unclear	1	0.037	0.028	0.046	<0.001		-
**Age range**							
Non-neonates	12	0.046	0.027	0.064	<0.001	0.001	97.6
Neonates	1	0.037	0.028	0.046	<0.001		-
**Region**							
Mainland China	6	0.033	0.013	0.052	<0.001	<0.001	95.3
Taiwan	5	0.067	0.041	0.094	<0.001		95.8
Hong Kong	2	0.013	0.007	0.017	0.761		0.0
**Study period, y**							
2001–2004	3	0.028	0.017	0.038	0.315	0.668	13.4
2005–2010	5	0.064	0.029	0.099	<0.001		98.9
2011–2014	3	0.033	0.013	0.053	0.001		85.7
**All studies**	**13**	**0.044**	**0.027**	**0.062**	**<0.001**		**97.4**

^a^The significance of the overall effect is calculated by computing a z-score as the ratio of the overall effect to its standard error and comparing it with the standard normal distribution.

^b^Two-sided z test was used to test difference of subgroups.

### Influencing Factors

Among the 13 articles, three articles examined influencing factors for MRSA nasal colonization among Chinese children. The significant influencing factors were identified through univariate analysis and they included gender (male vs female: OR: 0.67; 95% CI: 0.55–0.82), contact with younger age (OR: 2.98; 95% CI: 1.31–6.96; OR: 1.56; 95% CI: 1.21–2.00), attending day care centers (DCCs) (OR: 2.97; 95% CI: 1.28–6.76), having infectious diseases (OR: 2.31; 95% CI: 1.10–4.52), usage of antibiotics (OR: 2.77; 95% CI: 1.45–5.05), residing in northern Taiwan (OR: 1.41; 95% CI: 1.15–1.71), passive smoking (OR: 1.30; 95% CI: 1.02–1.63) and, pneumococcal vaccination (OR: 1.22; 95% CI: 1.01–1.48). Other influencing factors are reported in Table 3.

**Table 3:** Influencing factors of MRSA nasal colonization in Chinese children reported in the selected studies.

Influencing Factors, Odds ratio (95% confidence interval)	Jinjian Fu, 2015, [16]	Hongxiang Guo, 2013, [17]	Chihjung Chen, 2011, [21]
**Personal factors**			
Gender (male vs female)	0.61 (0.26–1.40)	1.17 (0.68–2.06)	0.67 (0.55–0.82)*
Age, y (2.5–6.7 vs 7–12)	2.98 (1.31–6.96)*	-	1.56 (1.21–2.00)*
Attending day care centers after school (yes vs no)	2.97 (1.28–6.76)*	-	1.19 (0.89–1.57)
Using antibiotics in a year (yes vs no)	1.71 (0.75–3.99)	2.77 (1.45–5.05)*	1.06 (0.87–1.28)
History of infection in a year (yes vs no)	2.03 (0.80–4.77)	2.31 (1.10–4.52)*	-
Having skin allergic diseases (yes vs no)	1.37 (0.52–3.28)	-	-
History of receiving outpatient service in a year (yes or no)	2.74 (0.96–7.50)	-	-
History of surgical operation in a year (yes or no)	0.00 (0.00–7.04)	2.35 (0.99–4.96)	-
Mode of production (normal childbirth vs Caesarean birth)	-	1.18 (0.61–2.19)	-
History of hospitalization (yes vs no)	-	1.21 (0.62–2.23)	-
Residing in northern Taiwan (yes vs no)	-	-	1.41 (1.15–1.71)*
Breast feeding (yes vs no)	-	-	0.99 (0.73–1.37)
Sleeping with parents (yes vs no)	-	-	1.11 (0.92–1.35)
Passive smoking (yes vs no)	-	-	1.30 (1.02–1.63)*
Pneumococcal vaccination (yes vs no)	-	-	1.22 (1.01–1.48)*
Flu vaccination (yes vs no)	-	-	1.30 (0.96–1.73)
History of acute otitis media (yes vs no)	-	-	1.12 (0.92–1.37)
Upper respiratory tract infection within 2 weeks (yes vs no)	-	-	0.98 (0.66–1.41)
Premature birth (yes vs no)	-	-	1.07 (0.71–1.56)
**Family factors**	-	-	-
Family members using antibiotics in a year (yes vs no)	1.93 (0.84–4.70)	-	-
Family members’ history of skin infection in a year (yes vs no)	0.73 (0.14–2.42)	-	-
Family members’ history of hospitalization in a year (yes vs no)	2.01 (0.50–5.98)	2.04 (0.76–4.65)	-
Family members are medical stuff (yes vs no)	1.58 (0.39–4.69)	2.35 (0.60–6.77)	-

* Statistical significant odds ratios.

### Publication Bias

Funnel plot for MRSA nasal colonization prevalence was displayed. According to the funnel plot, the studies were within the confidential interval and the shape of the funnel plot did not reveal any evidence of obvious asymmetry (S1 Fig). Additionally, Begg’s test and Egger’s test were performed to quantitatively evaluate the publication biases. According to the results, all the *p* values of Begg’s test (z = 0.67, *P* = 0.502) and Egger’s test (t = 1.03, *P* = 0.325) were above 0.1. Therefore, there was no strong evidence of publication bias and the results were reliable.

### Sensitivity Analysis

**Fig 3:**
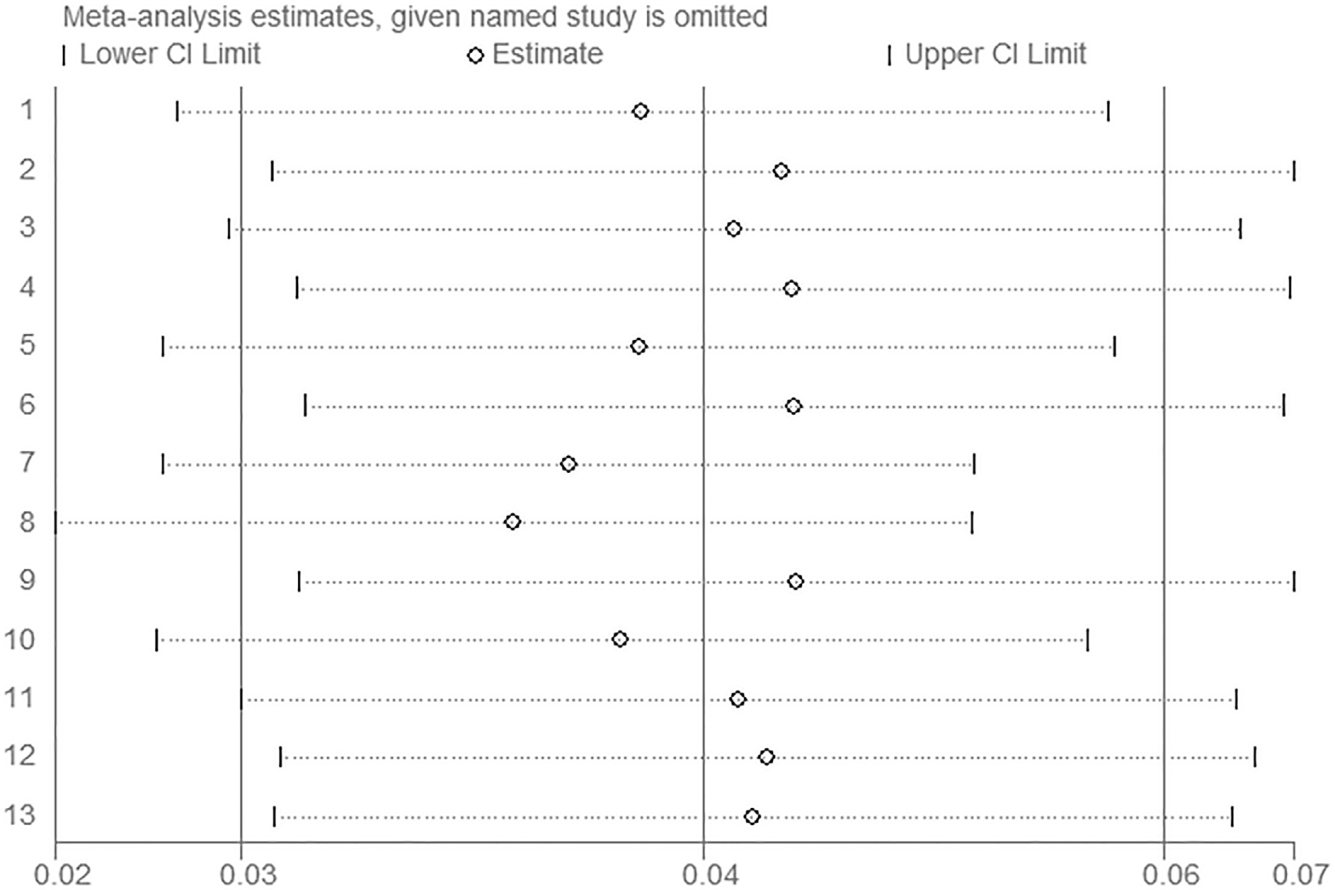
Sensitivity analysis of MRSA prevalence. (A) Results were computed by omitting each study in turn. (B) The two ends of the dotted lines represent the 95% CI.

To evaluate the contribution of a single study on the overall pooled prevalence and 95% confidential intervals, we performed sensitivity analysis by omitting individual studies one by one. The sensitivity analysis indicated that none of the individual studies greatly influenced the overall pooled prevalence. The leave-one-out prevalence estimate ranged from 0.037 (0.020, 0.054) to 0.048 (0.029, 0.066), suggesting that the results were consistent (Fig 3).

## Discussion

MRSA nasal colonization has been widely investigated in a variety of populations, resulting in heterogeneous prevalence and influencing factors. We performed this meta-analysis to summarize prevalence figures and influencing factors obtained from China, with an exclusive focus on the children. An overall pooled prevalence of MRSA nasal colonization was 4.4%, with higher pooled prevalence of MRSA nasal colonization among children recruited in the hospitals (6.4%) than those recruited in the communities (2.7%). When classifying articles by age range, pooled prevalence of MRSA nasal colonization was 4.6% in non-neonates and 3.7% in neonates. When stratified articles by region, children from Taiwan presented higher pooled prevalence of MRSA nasal colonization (6.7%) compared with those from mainland China (3.3%) and Hong Kong (1.3%). When analyzed articles by study period, the overall tendency was rising and there was a plateau in study period 2005–2010. As for subgroup of presence of underlying condition, pooled prevalence of MRSA nasal colonization was 3.9% in healthy children, 10.3% in children with atopic dermatitis, 12.5% in children with *S*. *aureus* infection, 1.0% in children with respiratory infection, and 3.7% in children with other underlying conditions. Several influencing factors of MRSA nasal colonization were also detected.

Our study indicates that the overall pooled prevalence of MRSA nasal colonization among Chinese children is 4.4%, which is much higher than that reported in other countries and areas [28], but is lower than that in Chinese adults [29, 30], which might also indicate that Chinese children are important reservoirs of MRSA and may play a central role in disseminating MRSA in the community and hospital settings. The regional distinctions are also observed by the result of our subgroup analysis stratified by region and supported by the findings of Chen CJ *et al* [21], which identified residing in northern Taiwan is a risk factor of MRSA nasal colonization. This phenomenon may result from variations of genetics or infection control policies, which need further explorations.

We noticed higher pooled prevalence of MRSA nasal colonization in children recruited in the hospitals compared with those in the communities, which indicates the hospitals remain the settings where the microorganism circulates most. This premise is in agreement with the results of a previous study, which demonstrates hospital-acquired MRSA isolates as dominant types in China [31]. Additionally, DCC attendance is recognized to be a significant risk factor for community-associated (CA)-MRSA in our study. Due to the crowded environment [21] and frequent close contacts among the attendees [16, 21], DDCs are favorable environments for transmission of *S*. *aureus* and MRSA, which was also reported in other countries [32, 33].

Moreover, our study demonstrates that children who were affected with underlying medical conditions, especially atopic dermatitis and *S*. *aureus* infection were easier to be colonized by MRSA and this phenomenon is consistent with several studies [34, 35]. However, due to the limited information of the included articles, we can not elucidate the concrete relation between MRSA nasal colonization and immunocompromized children, which is one of the limitations in this meta-analysis. Our subgroup analysis indicated that non-neonates have higher risk of MRSA nasal colonization than neonates. While, the influencing factors analysis demonstrated that younger age is a predictor of MRSA nasal colonization among children [16]. The controversial findings may partly explained by the very limited subjects of neonates in our study and the role of age on MRSA nasal colonization prevalence requires further studies. One of the three studies [16, 17, 21] concerning the relation between antibiotic usage and MRSA nasal colonization among healthy children revealed that antibiotic usage was independently associated with increased rate of MRSA nasal colonization [17], thus, health care providers (practitioners and specialists) should be made aware and encouraged toward a wiser usage of antibiotics for these groups of patients, taking into account the link between antibiotic prescription and emergence of antibiotic resistance, as demonstrated by several ecologic studies and clinical trials [36–38]. Having infectious diseases, smoking, and pneumococcal vaccination have been regarded as independent predictors of MRSA nasal colonization [17, 21], and their roles were confirmed by recent reports [39–41].

Finally, pooled prevalence of MRSA nasal colonization reached a plateau in the study period 2005–2010, which may result from the changing measurement of infectious control and various antibiotics usage [42–44]. Consequently, the association between MRSA nasal colonization and preventive measures as well as use of antibiotics should be more widely investigated.

According to the discussion above, relevant departments should pay more attention to the significant influencing factors and the significant results of subgroup analyses when they establish public health interventions to reduce MRSA infection. Relevant departments should pay more attention to children with female gender, younger age, attending DCCs, having infectious diseases, using antibiotics, passive smoking, and pneumococcal vaccination.

The meta-analysis has some merits. Firstly, all of the included studies have provided adequate number of population. Secondly, all the subjects are Chinese patients, thus ruling out the impact of ethnicity, which was thought to be a major potential confounder. Thirdly, the studies are published between 2005 and 2015, which are representatives of the epidemiology of MRSA in recent years. Fourthly, all the included studies were of high quality (score >5). Finally, it is known that MRSA is a serious threat to hospitalized patients globally and now represents a challenge for public health, as community-acquired infections appear to be increasing [45] in both adults and children across various regions and countries, including North America [45–49], Australia [50], Saudi Arabia [51], Finland [52], New Zealand [53] and, the United Kingdom [54]. This study is the first to systematically investigating MRSA nasal colonization prevalence in Chinese children and will provide epidemiological information of MRSA.

Nevertheless, our meta-analysis has some limitations. Firstly, a significant heterogeneity regarding MRSA nasal colonization prevalence among the studies included was observed. Differences in study populations, age range and regions might be the leading causes of such heterogeneity. Moreover, different method of MRSA identification and history of antibiotic usage in the included articles might also be the cause of such heterogeneity. However, analyzing data using the subgroup and sensitivity analysis did not significantly reduce the heterogeneity within studies. Secondly, due to the limited information of the included articles, we can not elucidate the concrete relation between MRSA nasal colonization and immunocompromized children. Another limitation is that we only focused on nasal colonization. At the moment, nostrils are recognized as an insufficient site for detecting a carriage status [55]. Other sites such as pharynx or perineum should be taken into account. Finally, the included studies were only dispersed in five provinces, four provinces lie in South China and only one lie in North China, which may not comprehensively represent the population distribution of the whole country.

In conclusion, the overall pooled prevalence of MRSA nasal colonization within Chinese children was 4.4%. More stringent MRSA prevention strategies are required in both hospitals and communities. In addition, clinicians should specially focus on the protection of children with gender of female, younger age, attending DCCs, having infectious diseases, using antibiotics, passive smoking, and pneumococcal vaccination to avoid severe adverse clinical outcomes.

## Supporting Information

S1 Fig Funnel plot of MRSA nasal colonization prevalence in Chinese children. (TIF)

S1 Table PRISMA Checklist of this meta-analysis. (DOC)
