# Supplementary figures and images for: Methicillin-Resistant Staphylococcus aureus Nasal Colonization in Chinese Children: A Prevalence Meta-Analysis and Review of Influencing Factors
Source: PLoS One. 2016 Jul 21;11(7):e0159728. doi: 10.1371/journal.pone.0159728 (PMC4956239; doi:10.1371/journal.pone.0159728)

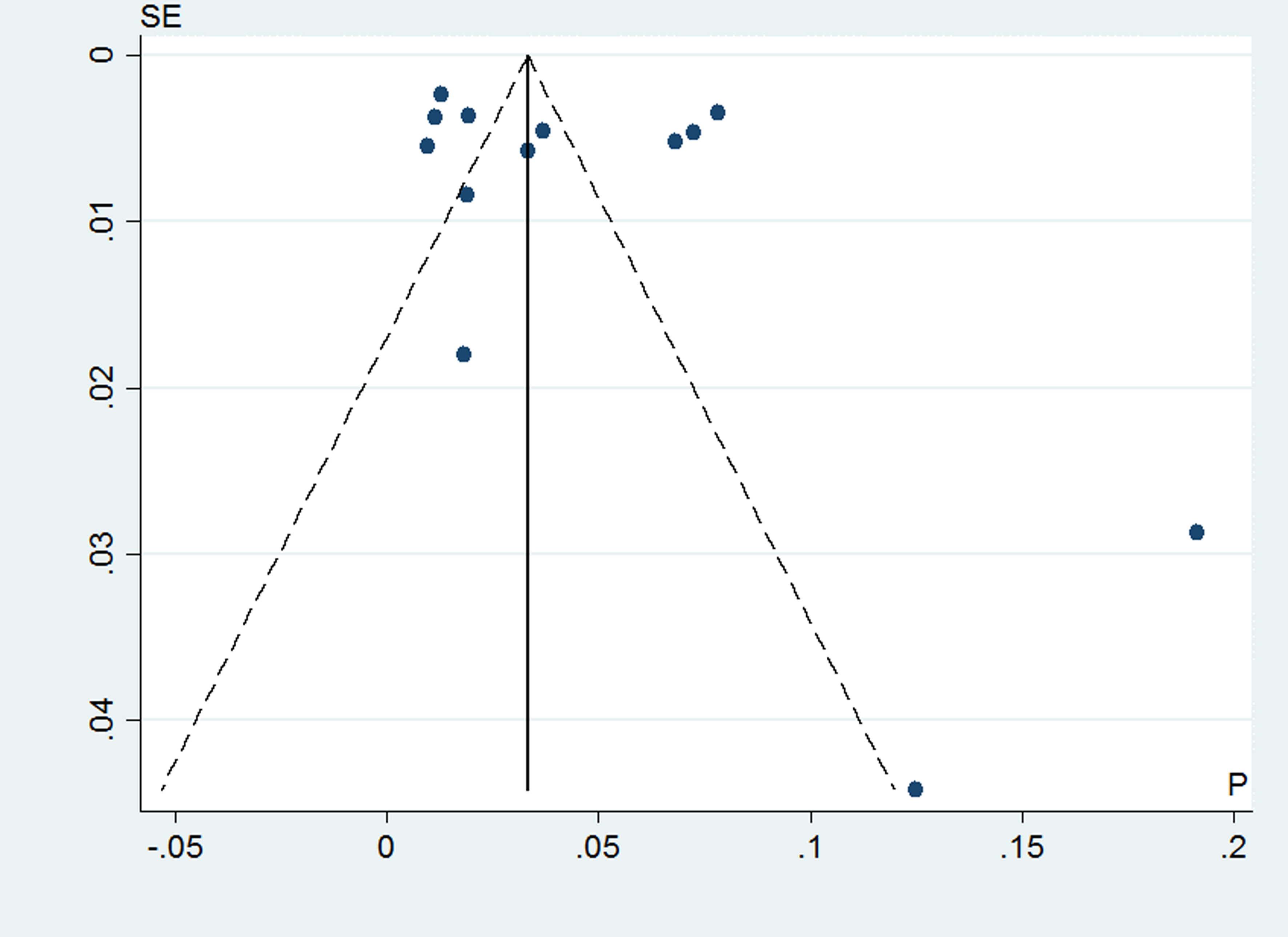

Supplement: S1 Fig — (TIF) [file pone.0159728.s001.tif]
